# Qualitative study of UK health and care professionals to determine resources and processes that can support actions to improve quality of data used to address and monitor health inequalities

**DOI:** 10.1136/bmjopen-2024-084352

**Published:** 2024-09-05

**Authors:** Sowmiya Moorthie, Emre Oguzman, Sian Evans, Carol Brayne, Louise LaFortune

**Affiliations:** 1 University of Cambridge, Cambridge, UK; 2 PHG Foundation, Cambridge, UK; 3 Hertfordshire County Council (HCC), Hertford, UK; 4 Hertfordshire Partnership University NHS Foundation Trust, Hatfield, UK; 5 Local Knowledge and Intelligence Service (LKIS) East, Office for Health Improvement and Disparities, Cambridge, UK

**Keywords:** health equity, health policy, qualitative research

## Abstract

**Abstract:**

**Introduction:**

Health inequalities in the UK are investigated and addressed by analysing data across socioeconomic factors, geography and specific characteristics, including those protected under law. It is acknowledged that the quality of data underpinning these analyses can be improved. The objective of this work was to gain insights from professionals working across the health and care sector in England into the type(s) of resource(s) that can be instrumental in implementing mechanisms to improve data quality into practice.

**Design:**

Qualitative study based on semistructured interviews involving health and care professionals.

**Setting:**

England.

**Participants:**

A total of 16 professionals, mainly from the East of England.

**Results:**

Awareness of mechanisms that could be put in place to improve quality of data related to health inequalities was high among interviewees. However, logistical (eg, workforce time, capacity and funding) as well as data usage (eg, differences in data granularity, information governance structures) barriers impacted on implementation of many mechanisms. Participants also acknowledged that concepts and priorities around health inequalities can vary across the system. While there are resources already available that can aid in improving data quality, finding them and ensuring they are suited to needs was time-consuming. Our analysis indicates that resources to support the creation of a shared understanding of what health inequalities are and share knowledge of specific initiatives to improve data quality between systems, organisations and individuals are useful.

**Conclusions:**

Different resources are needed to support actions to improve quality of data used to investigate heath inequalities. These include those aimed at raising awareness about mechanisms to improve data quality as well as those addressing system-level issues that impact on implementation. The findings of this work provide insights into actionable steps local health and care services can take to improve the quality of data used to address health inequalities.

**STRENGTHS AND LIMITATIONS OF THIS STUDY**

- Semistructured interview questions enabled collection of rich data on health and care professionals’ perspectives on actions to improve quality of data in relation to health inequalities.
- Inclusion of a range of participants across health and social care in the East of England.
- The study is limited by not being able to interveiw the full spectrum of those involved in health inequality data to decision pathway(s).

## Introduction

 The COVID-19 pandemic has highlighted the long-standing inequalities in health in the UK.^1^,^3^ There is now a renewed emphasis on the need for action to address these at both the national and system levels.^4 5^ This is reflected in the prioritisation of health inequalities in National Health System (NHS) England’s Long-Term Plan^5^ and in assigning Integrated Care Systems (ICSs) responsibility to proactively reduce health inequalities.^6^

Identifying the locations where health inequalities occur, establishing mechanisms to address them and assessing the impact of interventions depends on good-quality data.^7 8^ Criteria on what constitutes ‘good’ can vary, but essentially data need to be fit for purpose. This can be determined by considering characteristics such as completeness, accuracy, relevance, availability and timeliness.^9^ Many health-related datasets exist in the UK, and analysis of this data across socioeconomic factors, geography and specific characteristics including those protected by law such as sex, ethnicity or disability and socially excluded groups assist in endeavours to address health inequalities. However, the health data landscape is complex, with variations in data that are collected, its flow across the health and social care system and accessibilty.^10^ Furthermore, many datasets either do not routinely collect important information that can assist in identifying, monitoring and addressing health inequalities or are limited by poor quality.^11 12^ This means that available data may not always be used to the best extent.^13^ Action to ensure datasets are complete and timely was identified as one of five key priorities for the NHS in 2021/2022.^14 15^ At present, there is limited guidance available for those working in local health and care services on best practice approaches to improving the quality of data used to address health inequalities.

We previously undertook a scoping review of published scientific and grey literature to identify evidence-based approaches to improving the quality of data used to monitor and address health inequalities.^16^ A variety of actions to improve data quality were identified. While most of the studies we identified focused on ethnicity data, the findings were generalisable across other characteristics such as sexual orientation and gender. The identified mechanisms worked across different points of the data to decision-making pathway, and it was evident that often a multilayered approach is needed to ensure data quality is fit for purpose. For example, actions such as mandating data collection and implementing legal safeguards to ensure non-discrimination were important actions upstream of data collection that impacted on data quality by influencing the ability to collect data of a sensitive nature. Staff training to ensure understanding of what data is required and why ensures more effective data collection and completion. Following data collection, approaches such as linking and imputation could be employed to ensure completeness and enable use for analysis and insight.^16^

Given the renewed emphasis on efforts to address health inequalities at both a national and a system levels, and the significant role data can play in this context, it is crucial to understand how systems can easily implement the mechanisms described in our review. The objective of this research was to gain insights from professionals working across the health and care sector in England into the types of resources that can be instrumental to implement mechanisms to improve data quality.

## Method

### Study design

We conducted qualitative research using semistructured interviews to gain insights into the perspectives of professionals working in local health and care and public health systems. The interviews were conducted to investigate their understanding of mechanisms to improve quality of data used to investigate health inequalities and uncover effective strategies to communicate and transition the evidence from our scoping review^16^ into practice. The interviews utilised open-ended questions, and a topic guide (online supplemental file 1) was designed prior to interview to guide data collection. The Standards for Reporting Qualitative Research reporting^17^ guidelines were used where applicable (online supplemental file 2). The study was designed and conducted by a research team that included both academic and service partners.

### Sampling and recruitment

Participants included in the study were professionals that had a role in public sector health and social care, public health or third sector organisations in England. Participants had to have as part of their role a responsibility to (1) address health inequalities and/or (2) collect and/or analyse and/or monitor and/or report data that can be used to understand health trends and inequalities at the organisation or regional level.

We worked with key contacts within the National Institutes for Health and Care Research NIHR Applied Research Collaborative East of England (NIHR ARC EoE) and employed purposive sampling using information available in the public domain to recruit relevant and, where appropriate, a sample representative of different roles. This allowed compilations of an initial list of individuals in collaboration with our service partners. In addition, we used a snowballing approach to identify further participants. To ensure participants were able and willing to contribute to the study, eligibility was ascertained by personal communication prior to data collection.

### Data collection

Data collection was undertaken through interviews conducted and recorded online via Zoom to provide greater time and location flexibility for participants. This enabled contributions from participants across the East of England. In the participant information sheet, participants were offered in-person or online options. All participants opted for online. Interviews were conversational, thus enabling participants to answer broad questions around their role, understanding of health inequalities, mechanisms to improve data quality and resources or tools that could enable improvements in data quality. Interviews lasted between 45 min and 60 min and two members of the research team, one academic (SM) and one practice-based (EO) undertook each interview to reduce bias and ensure robustness in data collection. Interviews were conducted between January 2023 and May 2023. Data were analysed continuously and recruitment stopped once data saturation was reached.

### Analysis

Interviews were transcribed by the research team (EO and SM) and transcripts anonymised to remove any identifying features. Thematic analysis was carried out using NVivo V.12 software. A deductive approach to coding interview data was initially taken based on the topic guide to categorise the interview data. Three interviews were coded independently by two of the team (SM and EO), coded data were discussed and reviewed. A common coding framework was developed and applied to remaining transcripts. Each transcript was doubly coded, and any discrepancies were resolved by discussion. The coded data were then analysed, an inductive approach used to identify themes and links across themes was identified to extend the findings and help structure and summarise the findings into a coherent and practice-focused narrative. We tabulated frequencies of theme elements to enable visualisation of findings. 

### Patient and public involvement

Patients and the public were not involved in the design and conduct of this study.

## Results

### Participants

The characteristics of the 16 interview participants were organised in terms of their organisational representativeness, regional coverage, knowledge of health inequalities and relationship to the data-to-decision-making pathway (table 1). Most participants worked in an organisation from a county within the East of England (87.5%, n=14). All but one participant (93.75%, n=15) expressed some knowledge of health inequalities. Over half the participants worked in the NHS (62.5%, n=10) and were involved in an analyst role (68.75%, n=11).

**Table 1:** Participant characteristics

	Number of participants
Organisation	
Local authority	3
NHS	10
Other	3
Area	
Suffolk and North East Essex	2
East Suffolk and North Essex	1
Norfolk	1
Hertfordshire	6
Bedfordshire and Milton Keynes	1
Cambridge and Peterborough	3
Other	2
Knowledge of health inequalities	
Yes	15
No	1
Role	
Data analysis	11
Data collection	1
Data intelligence user	4

NHSNational Health System

### Thematic analysis

Findings are grouped under three themes that were drawn from the topic guide. These were:

1. Awareness of actions to improve data quality.
2. Challenges in implementation of actions to improve data quality.
3. Resources that can aid implementation of actions to improve data quality.

### Theme 1: Awareness of actions to improve data quality

This theme describes participants’ awareness of mechanisms to improve data quality and which, if any, they have implemented. It also considers the resources used or created to enable improvements in data quality. This question was posed to participants to gain a better understanding of the current state of practice regarding mechanisms to improve data quality.

Most participants we interviewed were aware of mechanisms that could be implemented to enable improvement in data quality (n=13). Two participants acknowledged their awareness of data quality issues, but not of mechanisms for improvements.

**Figure 1:**
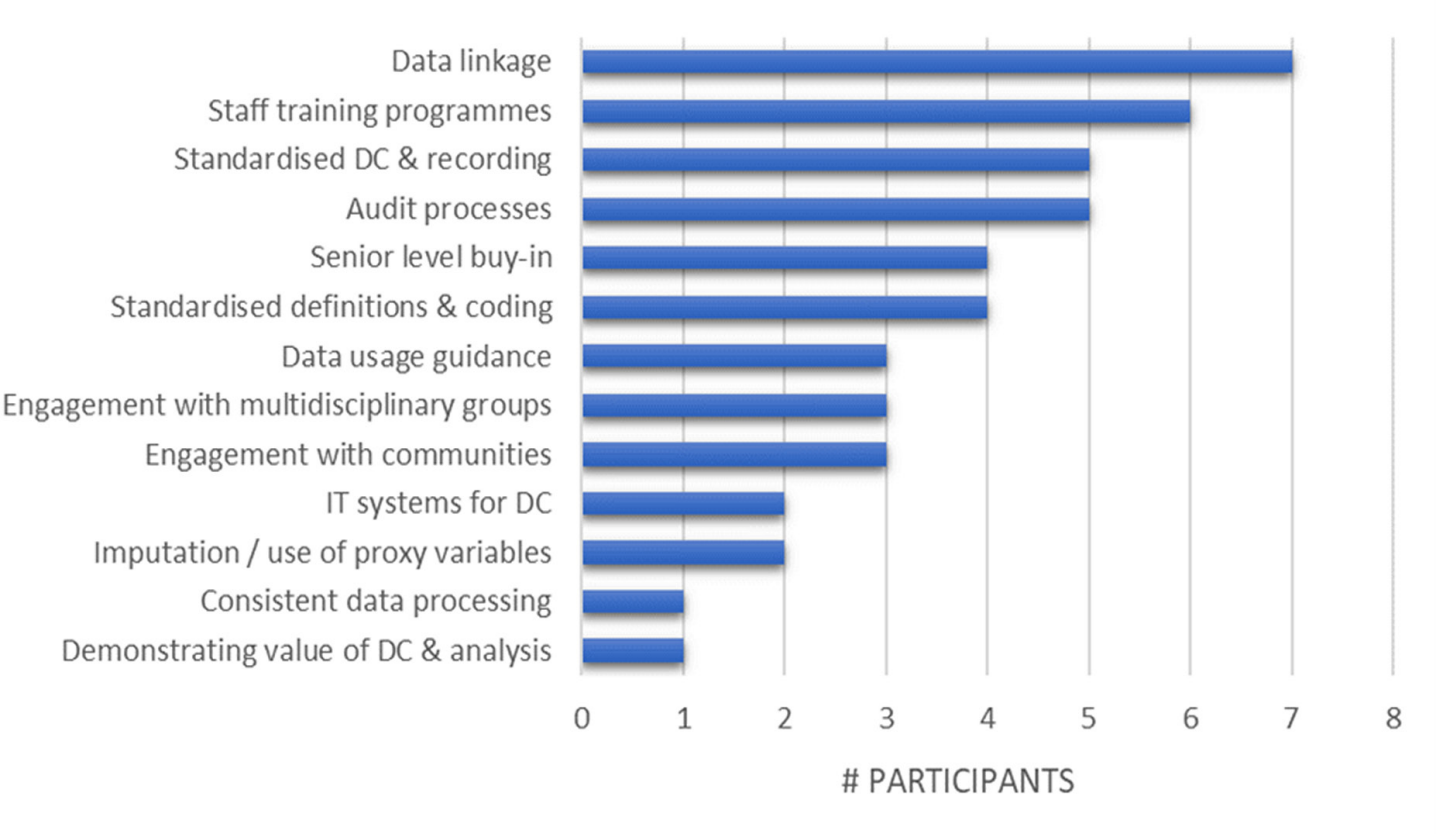
Mechanisms to improve data quality. DC, data collection.

Participants cited many of the actions that we had identified in our underlying scoping review,^16^ along with specific mechanisms in place to achieve them. The most cited actions to improve data quality were data linkage and staff training programmes (figure 1). The least cited were mechanisms to demonstrate the value or impact of data collection on service provision to a wider audience, for example, senior leaders or the public and patients. Processes that were implemented to maintain the accuracy and completeness of datasets obtained from sources both within and external to participants’ organisations were also not widely cited.

The extent to which actions were put into practice varied by organisation (eg, NHS vs council) and in the specifics of the mechanisms described. For example, in relation to staff training programmes, some described the creation of a health inequalities intranet with educational resources, others described approaching this through awareness raising mechanisms such as forums and staff networks. Such resources were developed to improve staff understanding of health inequalities and rationale in collecting data on protected characteristics such as ethnicity.

I think locally what we try to do, for those who are collecting data and sometimes when you speak to GP practices, or at least in the past when we did this. Once you have explained how this information could be used, and the importance of it being right first time. People actually made more of a conscious effort then to make sure it was right the first time, and I think educating or explaining to people who are working, collecting that information and inputting it in the first place, you can put quite a lot of checks in. P3

And because we realized that, you know, people staff are quite uncomfortable asking about ethnicity…. So we gave people a card which we did co-produce a bit with XXX public health, didn't we? …. But a really simple card that had the categories on one side and then on the other there was some basic answers. If people asked why this was needed, a really simple data Protection type level about kind of what you're gonna use this for. P1

This reflected organisations’ development of their own mechanisms depending on specific priorities, needs and circumstances. For example: data needs; stage of organisations in creating and using integrated datasets; stage of developing information governance practices to enable linkage and analysis. These factors influenced the specific resources that were used, created or needed.

### Theme 2: Challenges in implementing mechanisms to improve data quality

**Figure 2:**
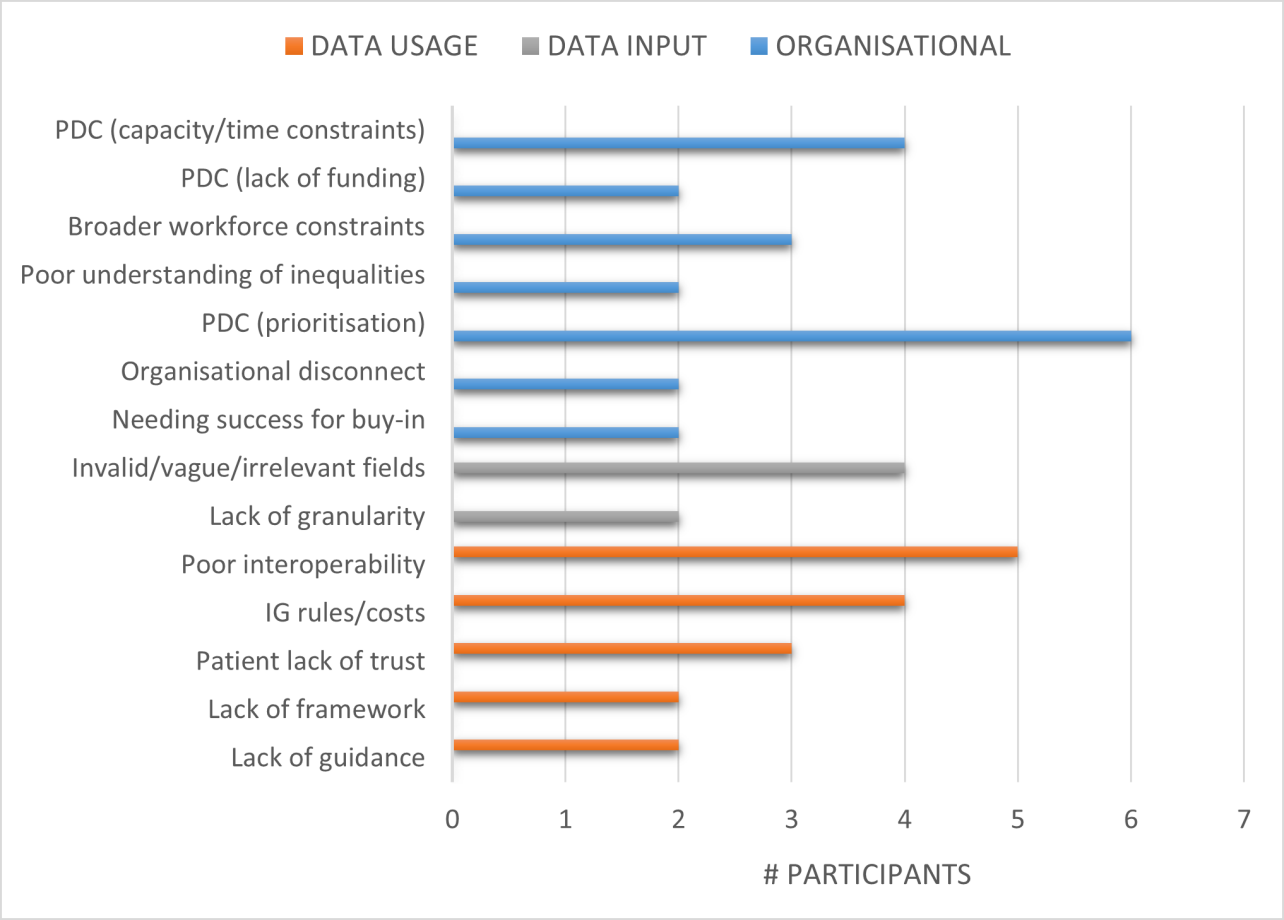
Challenges in implementing mechanisms to improve data quality. PDC: poor data collection, IG: information governance

Factors that were felt to impede efforts to implement actions to improve data quality and undertake work on health inequalities were gathered under this theme. These were further categorised into three broad categories of either organisational issues that resulted in poor data collection, data input or data-related issues that created challenges in optimal data usage and analysis (figure 2).

#### Organisational issues that impact on data collection

Participants identified logistical barriers to implementing mechanisms to improve data quality within their organisations. These included workforce time, capacity and funding constraints and team structures that impacted on cross-team or cross-organisational working. A quarter of participants stated that the quality of data deteriorated when data collectors faced time constraints and reduced capacity (n=4). Some NHS professionals attributed poor data collection to lack of incentives and funding (n=2). There was the perception of a *bias* that larger NHS organisations, due to their greater financial resources, have reporting infrastructures that can adequately capture and record more data, whereas this was not possible for smaller organisations. These workforce time, capacity and funding constraints were not found to be exclusive to the collection stage. Similar issues were cited as impacting analysis, outreach activities across the system and reporting for work on health inequalities (n=3).

Among some NHS professionals, there was a perception that health inequalities were not fully understood within the health system (n=2).

I think health inequalities is poorly understood in the main, I think, for those people that sort of live and breathe it and or actively sort of talking about it. They understand it. But actually, if you talk about health inequalities with frontline clinicians, their view is likely to be very, very different, and I think that’s the biggest hurdle to overcome, and it’s just trying to almost educate the wider workforce around. Why, it’s important for this topic to be understood more broadly. P15

This lack of deep understanding was argued to produce data reporting structures that provided limited information on patient demographics. For example, half of participants related poor data quality to data collectors not knowing what to ask (n=6). This was most often viewed as an issue of prioritisation, where organisations had set collection standards for only a few variables, particularly ethnicity, while neglecting others. One participant noted that there were no established targets for physical disability within their organisation, and so ‘*nobody bother(ed) collecting that data’*.

There was recognition that putting in place mechanisms to improve data quality required leadership and involvement of those across the data-to-decision-making pathway. Some participants described a disconnect between organisational groups working at different stages of the pathway (n=2). Some postulated that those who used intelligence from data such as managers or executives had a better appreciation for a need to implement actions to improve data quality, as they could see the impact of good data on improved care. As one participant in social care reflected:

I think there might be a disconnect, maybe between the people at the top that probably see the value of it, and the people in the frontline that probably don’t have the capacity to be able to collect that information. P5

Conversely, some data intelligence users suggested that senior-level buy-in was typically achieved by reporting the success of work on health inequalities (n=2). This was reported to be a ‘*difficulty’* and a ‘*challenge’* because addressing health inequalities was viewed as a long-term task and immediate impact was hard to evidence. These users felt that they had to provide answers to senior groups and leaders within a short timeframe and show that any actions they implemented were *doing the right thing*.

#### Data usage barriers

Issues related to the use and handling of data were also described, namely with reference to datasets. This included the existence of invalid, vague or irrelevant observations in datasets (n=4). These features were recognised as irreparable, despite the availability of quality improvement tools, such as postcode look-ups, for example. In the context of using UK postcodes to measure social deprivation, one analyst noted:

(With postcodes), if they don’t have a space, it’s not such an issue for us (…) we have lots of different lookups that we use to (add or remove spaces) (…) We have issues when someone’s entered a postcode that doesn’t exist. And it’s like, well, you can’t really do anything with that… P1

Two participants cited lack of granularity, where information for health inequality variables exists but is not sufficiently detailed. For example, there was an acknowledged awareness and use of national and interorganisational datasets that had been compiled and distributed for analytical work on health inequalities. However, participants could not exploit datasets in some cases due to data over-aggregation and the presence of broad variable categories. For example, the aggregation of data across large geographical areas such as local authority level, when it was often desired at the district level. Aggregation of ethnicity data into categories such as ‘Asian’ as opposed to ‘Pakistani’, ‘Indian’ etc. These factors were deemed to hinder work on identifying target populations or geographic areas of concern within participants’ region of operation.

Participants also stated that wider interorganisational structures and practices impacted on the ability to link and make use of existing data. This included poor data interoperability between healthcare organisations and systems (n=5) and inconsistency in patient records between different organisations, including community services, the NHS and local authorities. Some attributed the inconsistency to the *complexity of service delivery*. One participant noted how screening services were provided by several community providers who collect, process and release data through different processes and at varying time points. Despite success in gathering data from these providers, the ability to mobilise this data to address health inequalities was impeded due to fields not being coded congruously.

Information governance rules and costs were also perceived as a barrier for data linkage (n=4). Many participants recognised the value of data from primary care, which was seen as having a higher level of granularity that was not found within local authority or secondary care datasets. They expressed frustration with having to first persuade general practitioners (GPs) to share data and then pay for extraction and linkage, often managed by a third party. It was reported by some participants that GPs may be concerned with data sharing because it was not an action that *patients actually agree to be part of explicitly*. Some participants cited a lack of trust on the part of patient groups as a barrier for data completeness (n=3). They perceived a poor relationship between patients and staff as an issue within their organisations; and that ethnic minority groups would be *more likely to decline providing an ethnicity* due to *fear or stigma*. This issue was also related to discrepancies in data completeness between organisations. As one participant commented:

I also know that people get fed up of always having to tick the boxes (…) ‘I already told my GP, why are you asking me again?’ And then they go to the community services and have to tick the box again. I totally understand why people might be like: ‘I’m not having to do this for the third time as part of my pathway. P16

The absence of a national framework on data sharing was found to be a barrier for data sharing and reporting (n=2). In the case of sharing, this meant that data linkage and accessibility was a *complicated* and *case by case* process bound by local organisation-specific information governance rules. One participant from social care attributed *“(reporting) inconsistencies across NHS trusts* to the absence of a national framework, which could propose guidelines for developing and reporting common measures.

Two participants argued that data were not appropriately used due to lack of data skills such as analytical abilities to understand the data and devise suitable metrics. In cases where the skillset was present, some found that there was little guidance on what variables they should consider during analysis (n=2). One participant had knowledge of relevant methodologies, such as benchmarking or using proxy measures, to investigate hard-to-reach or disadvantaged populations. But this was only applied for ethnicity, and guidance was needed on other variables, such as homelessness and disability.

### Theme 3: resources that could aid improve quality of data

Participants were asked to provide examples of resources that are particularly helpful or lacking in efforts to either raise awareness of mechanisms or put in place actions to improve data quality. This was to understand current availability of resources and any gaps or needs that could be addressed.

#### Currently available resources and gaps

Some participants acknowledged that a wide variety of resources and tools were already available that could aid in efforts to improve data quality. These ranged from information sources on health inequalities and explanation of indicators. These can help by improving awareness around health inequalities and data collection efforts. In addition, for those involved in analysing data, packages that can aid in data analysis and forums for discussion of analytical approaches were available. However, participants also indicated that these resources were created by different groups and organisations, therefore not collated or catalogued and accessible from a single reliable source.

I think that would be helpful to kind of have the one place to go to where you could get like you say, all the information and kind of different steps that you could take all the other people have taken which I don't really well, doesn't exist at the moment. So if it does exist, maybe it needs to be public. P1

This meant that while many resources were available, finding them and ensuring they were appropriate were time-consuming. One participant expressed difficulty in choosing a resource from several available options. They found that it was *difficult to navigate what’s new and what’s a repeat* during interorganisational meetings.

#### Desired resources

As participants who were interviewed were for the most part aware of mechanisms that could be put in place to improve data quality, the resources they requested were in supporting delivery of these efforts. Specific suggestions that were put forward by participants were grouped under four categories (table 2) and are discussed below.

**Table 2:** Categories of resources suggested by interview participants

Category of resource	Suggested focus of resource
National guidance, standards and frameworks to support action along the data to decision pathway	Creating a shared understanding around health inequalities
	Best practice around categorisation of particular variables such as ethnicity
	Guidance or best practice on how to collect data
	National framework outlining consistent measures that can be used in relation to health inequalities
	Standard mechanisms for reporting and quality assurance around data categories
Sharing of what works/lessons learnt between organisations and systems	Strategies used to communicate with the public and health professional on how and what the data is being used for
	Mechanisms used to communicate the impact of data on health inequalities
	Approaches to navigating information governance structures across organisations
	Actions taken to improve data completion
	Examples of what has been done in other parts of the country to improve data quality
Communication across the data to decision pathway within organisations and groups	Creating a better understanding of analysis processes and shortfalls in what can be done with existing data
	Demonstration of contribution of different stakeholders to the health inequalities data pathway
	Cross-team communication to enable feedback to data sources/collectors on the gaps in data
Tools to aid health inequalities data analysis	Data catalogue or repository providing information/metadata on available data sets
	Best practice in terms of methodological approaches
	Increasing interoperability between data sets
	Improving knowledge of tools that can be used in data analysis, eg, Fingertips, or other tools for population health management.

National guidance, standards and frameworks to support action along the data to decision pathway

National guidance in different areas was put forward as being needed, in particular, for a shared understanding of health inequalities. Many interview participants cited the fact that ‘health inequalities’ was a broad term, with variation across the health and social care system on how it is viewed and conceptualised. This is also reflected by differences in particular aspects or questions in relation to health inequalities that are the focus of different organisations and groups or teams across the health and care system. Therefore, how health inequalities are conceptualised, impacts on data requirements and whether data quality is considered fit for purpose. In addition, while addressing health inequalities was a policy priority, there were differences, and sometimes a lack in understanding of what this meant, especially for many who do not directly work on tackling inequalities but nevertheless contribute to the data to impact pathway, for example, data collectors such as clinicians or high-level decision-makers such as hospital medical directors or those who sit on boards for ICSs. Therefore, a shared understanding around health inequalities can also aid individuals who are involved either directly or indirectly in the data to decision pathway implement actions and efforts to improve data quality.

Because how you see health inequalities from different lens, and everyone is contributing to it. But it is slightly different, and you know, and everyone have to play their part to bridge this gap. Having that clarity would really help people to do that. P8

Many participants, especially analysts, stated that while they were aware of mechanisms that can support actions to improve data quality, it was unclear what was best practice in relation to these. In particular, best practice in relation to the following was reported as being useful:

- Categorisation of particular variables such as ethnicity.
- Methodological approaches to data analysis.
- Data collection and ensuring data completion.
- Sharing of what works/lessons learnt between organisations and systems.

Many participants discussed how sharing of strategies and mechanisms employed by other organisations could inform the resources they develop or practices they undertake in improving data quality. This was perceived as being especially useful where organisations and/or systems were at different stages of achieving integrated data and taking different approaches to solving common issues. For example, navigating information governance processes across organisations was an oft-cited barrier (figure 2). In addition, incomplete data were also a common problem faced by different teams and organisations. Therefore, sharing approaches that had been taken in addressing common issues would be useful learning.

- Communication across the data to decision pathway within organisations and groups.

Participants also stated that communication across different individuals, teams and organisations was required to enable improvements in data quality. Many cited case studies to demonstrate the value of data and data-to-impact pathway as a means to enact change.

The more we sort of say, you know that the importance of using data to form evidence-based sort of outcomes, or projects and programs the more they’ll become more embedded in all programmes at work. P15

However, they also acknowledged that such case studies would have to be adapted to meet the needs of the different roles along this pathway and take into consideration different data to decision-making pathways.

Participants also stated that the role of data and its impact need to be better communicated. This is because achieving senior-level buy-in, encouraging better data collection and contribution from patients and the public requires demonstration of impact. Therefore, developing resources that could demonstrate the value of the data and its impact is important for incentivisation. Inclusion of information on how data is processed or analysed to address inequalities was also put forward as an important aspect. This was seen as being useful in addressing concerns on uses of the data and in improving understanding and trust of how data are handled and processed. It could also provide an opportunity to demonstrate how different stakeholders contribute to the data to decision-making pathway and in improving data quality. In addition, this was felt as particularly important for users of health intelligence, as it enables them to better understand how improvements in data quality can impact on producing informative intelligence.

I would really like to see if it’s available on a website that would be great, where you've got the sort of the whole process from data collection all the way through to potentially interpretation. So that then you can make decisions based on the information that comes out of it after processing all that data and doing all the analytics. And it'd be really nice to have that quality step along the entire analytical pathway. P4

#### Tools to aid health inequalities data analysis

Many participants stated that practical resources to aid analyses pertaining to health inequalities would also be helpful. For example, a data catalogue that had information on different data sources available, meta-data on the variables and suitability for use in different types of analysis were proposed. Participants also noted that there were similarities in the types of questions asked across organisations, therefore best practice guidance in answering common or routine questions, data that can be utilised for this, and caveats in available data would be useful.

## Discussion

Addressing and monitoring health inequalities continue to be an important policy goal that is reliant on good-quality data. A variety of mechanisms exist to improve the quality of data used for this purpose.^16^ This study was conducted to explore effective ways to improve knowledge and implement these mechanisms in practice.

Interviews with health and care professionals suggest that most are aware of the mechanisms we identified in our scoping review. We also evidenced examples of how these mechanisms had been embedded in practice. However, it was apparent that the evolving health and care data ecosystem in the East of England and nationally meant that different organisations were at different stages of addressing data quality issues and were taking different approaches to do so. It was recognised that more needed to be done and several barriers that impeded efforts to improve data quality were cited. This included logistical factors such as having a data-skilled workforce, capacity and functioning issues in a resource-constrained environment, and system-level issues such as creating interoperable data systems and information governance structures to support better data use. Many of these have been cited as issues in previous work on better use of data for healthcare.^18^,^20^

This study identified that different approaches were needed to both increase awareness of the variety of mechanisms that could be employed to improve data quality as well as support their delivery. When asked about resources that could support current practice, suggestions included developing national guidance, standards and frameworks to support action along the data to decision-making pathway. For example, some participants argued that the absence of a national framework was a perceived barrier for data sharing and reporting on health inequalities. Some national frameworks have considered improvements to the quality of health data.^21 22^ For instance, the NHS England operating framework asks National Programme teams to set expectations and guidance on data standards.^22^ This suggests that current national guidance, while useful in acknowledging the importance of data quality, may need to extend beyond data collection and collation to more comprehensive guidance on data usage and be promoted within organisations as much as possible.

It was evident from our interviews that individuals and teams often created their own resources to enable engagement on mechanisms to improve data quality. This is unsurprising given the complex data landscape.^10^ Participants stated that sharing strategies and mechanisms employed by other organisations such as different NHS Trusts across the country would be helpful. This includes strategies employed to improve data collection, information governance structures that work well and tools that can be used in data analysis, for example, in the specific context of population health management.^23 24^ Learning from these would be useful in enabling a more consistent approach. Case studies were suggested as one mechanism through which examples of approaches taken could be shared among different individuals, organisations and systems.

Overall, resources that can aid in creating a shared understanding around health inequalities and improving mechanisms to share knowledge between different systems, organisations and individuals were key. A better understanding of inequalities can also help frame and formulate appropriate questions, which in turn enables assessment of data quality parameters. Data quality is characterised by completeness, accuracy, relevance, accessibility and timeliness.^9^ While there was consensus that mechanisms could be put in place to improve factors such as data collection and granularity of data, there was also a recognition that for some purposes data quality was adequate. This understanding is also required to enable interpretation of intelligence gained from data analysis.

Differences in understanding of what health inequalities means both conceptually and from various practice perspectives have been raised by others as an issue that impacts on practical actions that are taken.^25 26^ Furthermore, the importance of framing inequalities in a way that is relevant for different organisations and individuals across the health sector has also been raised to enable more effective action.^27 28^ Understanding is also needed by the wider public, as it can aid in creating trust and a better understanding of how data are used. This is a critical building block to enable better data collection, especially around protected characteristics, and to address concerns around stigmatisation.^8 29 30^ The need for deliberation and engagement with the wider public to design the best approaches to collect sensitive data, as well as with data collectors to improve health inequality literacy, was one of the actions identified by our previous review to improve data quality.^31 32^

Improving communication between individuals across the data to decision pathway was also felt to be an important action to achieve improvements in data quality. This is because a variety of health professionals are involved across different pathways and differentially contribute to improving data quality. For example, data collection is often undertaken by front-line staff, whereas analysis and use of data intelligence will be led by other professionals. Some participants noted that users of data intelligence may have a better appreciation for a need to implement actions to improve data quality than collectors. This demonstrates the importance for organisations to introduce communication channels between both groups when absent, and support channels that have developed, to enable better collection and use of data. Indeed, efforts to improve data quality require input from a variety of individuals across the data to decision pathway, and data quality improvement strategies often require assessing this pathway.^33^

Most participants worked in an analytical role; hence resources to assist with data analysis were also deemed important. Suggestions put forward, were repositories that could support data analysis such as those providing information on suitable data sets and methodological approaches that could be applied in data analysis. In addition, while many participants were aware of common knowledge sharing platforms (eg, FutureNHS) and data collections (eg, Fingertips), there is still a need for improving this awareness across groups and organisations.

### Strengths and limitations of this study

Using a qualitative approach has allowed us to explore the needs of health and care professionals and identify key features and types of resources that can aid in embedding practices to improve data quality. Our approach of using open-ended questions in the interviews, allowed participants to speak openly and enabled gathering of rich data. Data coding was carried out independently initially, and following development of a coding frame, all transcripts were double-coded to ensure consistency. Interpretation of findings were discussed among the team and further validated by sharing with interviewees.

The study included a range of participants; however, we were not able to interview the full spectrum of roles involved in health inequality data to decision pathways. In particular, those involved in senior decision-making roles, data collectors and data managers were not represented in our sample. In addition, given the variety in data to decision-making pathways in existence, it is likely that the needs of each pathway may differ. Thus, our findings may not reflect the full spectrum of perspectives across the health and care system. Also most of our participants were based in the East of England region, potentially influencing generalisability of findings, especially to international contexts. Nevertheless, the core functions of any resources that would help embed evidence-based mechanisms to improve data quality may be applicable to a range of settings.

### Conclusions

This study identified the types of tools or resources that could help embed mechanisms to improve the quality of data used to monitor and address health inequalities. These resources would have to take a variety of shapes, given the diversity of health and care professionals who contribute to data quality improvement efforts. The development of case studies to engage with the broad range of individuals who contribute to the data to decision pathway was highlighted as a potentially useful resource. We are currently working on developing and testing the impact of such case studies. Finally, while good-quality data are important in efforts to investigate and address health inequalities, it forms part of a larger landscape of efforts needed.^27^

## supplementary material

10.1136/bmjopen-2024-084352 online supplemental file 1

10.1136/bmjopen-2024-084352 online supplemental file 2

## Data Availability

Data are available upon reasonable request.
