# Supplementary material for: Qualitative study of UK health and care professionals to determine resources and processes that can support actions to improve quality of data used to address and monitor health inequalities
Source: BMJ Open. 2024 Sep 5;14(9):e084352. doi: 10.1136/bmjopen-2024-084352 (PMC11381701; doi:10.1136/bmjopen-2024-084352)
Supplement: online supplemental file 1 [file bmjopen-14-9-s001.pdf]

Note: This is a basic topic guide for semi-structured interviews with professionals working in health and social care, public health, and third sector organisations. It will be adjusted as the study evolves. Questions will be tailored to individual participants and their expertise, and adapted for use in individual interviews and focus groups.

1. Interviewee background

Participant's awareness of inequalities in health and healthcare

Participant's professional role and involvement in the health inequalities data pathway.

Participant's involvement in data pathway (e.g. data collection, data coding and input, data analysis, interpretation of data and data informed decision-making) especially in relation to health inequalities

Participant's experience of quality improvement approaches

Relevance of a resource to participant's practice

2. Mechanisms to improve data quality

Are you aware of any approaches that focus specifically on improving the quality of data relating in health inequalities?

What existing tools, if any or resources do you use in improving data quality.

Are they sufficient and meet your needs?

Examples of resources/tools that are particularly helpful

Examples where resources/tools are lacking.

How could knowledge of distal initiatives to more proximal initiatives to improve data quality be better understood/communicated.
