# Supplementary material for: Qualitative study of UK health and care professionals to determine resources and processes that can support actions to improve quality of data used to address and monitor health inequalities
Source: BMJ Open. 2024 Sep 5;14(9):e084352. doi: 10.1136/bmjopen-2024-084352 (PMC11381701; doi:10.1136/bmjopen-2024-084352)
Supplement: Supplementary file 3 [file bmjopen-2024-084352.reviewer_comments.pdf]

PEER REVIEW HISTORY

BMJ Open publishes all reviews undertaken for accepted manuscripts. Reviewers are asked to complete a checklist review form and are provided with free text boxes to elaborate on their assessment. These free text comments are reproduced below.

ARTICLE DETAILS

**TITLE (PROVISIONAL)**	Qualitative study of UK health and care professionals to determine resources and processes that can support actions to improve quality of data used to address and monitor health inequalities
**AUTHORS**	Moorthie, Sowmiya; Oguzman, Emre; Evans, Sian; Brayne, Carol; LaFortune, Louise

VERSION 1 - REVIEW

**REVIEWER NAME**	Sealey, Clive
**REVIEWER AFFILIATION**	University of Worcester, Allied Health and Community
**REVIEWER CONFLICT OF INTEREST**	None
**DATE REVIEW RETURNED**	29-Jan-2024

**GENERAL COMMENTS**	The paper provides some relevant analysis of some key issues that are relevant to data concerned with inequalities. The discussion highlights some important recommendations for policy and practice. However, thee paper could be improved by attention to the following:<br><br>1. The Title of the study focusses on resources, but some of the issues identified are not resources but processes i.e. communication. Therefore could the title be changed resource and processes.<br><br>2. On page 4, it states that 'In addition, data that are available are not always used to the best extent'. This needs to be referenced.<br><br>3. On page 7, you say that the tried to recruit a representative sample. However, there is no indication of what the sample is representative of. This needs to be made clearer.<br><br>4. On page 6, it states that the interviews were conducted via Zoom, but no rationale is provided for this.<br><br>5. On page 7, it says that the interviews were undertaken by 2 members of the research team. This needs to clearly state if by this is meant 2 members together, or 2 members individually, and if the former, the reason for this.<br><br>6. On page 9, it states that an inductive approach to data analysis was used, however, closer analysis suggests that it was actually Inductive/Deductive Hybrid Thematic Analysis (as set out here: https://journals.sagepub.com/doi/10.1177/15586898221126816), which was undertaken. This needs to be clarified.<br><br>7. The Table of participants needs to be moved to where the sample frame is discussed as this is not results. Additionally, on page 8

	where you say working across health and social care, this needs to be more specific if it refers to public sector health and social care organisations, or both.<br><br>8. There are variations of thematic analysis, and it would be good for the specific TA approach used to be specified and rationalised.<br><br>9. It would be good to know what the role of the participant was without a knowledge of health inequalities. For example, if this refers to the data analyst, then this could be significant initself.<br><br>10. The tables presents for Analysis of Themes 1 and Themes are clear and detailed, but they do not particularly represent thematic analysis, they are more representative of quasi-statistical analysis, and this needs to be acknowledged and highlighted in the section on data analysis techniques.<br><br>11. Linked to this is the fact that these themes seem to indicate that either some form of quantitative data collection took place, or that the interviews had a highly structured element. This needs to be made clearer.<br><br>12. Data usage barriers - should this be subdivided in the first part as Data Input issues?<br><br>13. In general, the participant's quotes are used very minimally, which is the essence of qualitative research. For example, Data usage barriers needs more validation of data through the use of participant's quotes. This needs to be reconsidered throughout the paper.<br><br>14. Quote on page 19 is free hanging, needs some linkage.

**REVIEWER NAME**	Kumar, Ramya
**REVIEWER AFFILIATION**	University of Jaffna, Community and Family Medicine
**REVIEWER CONFLICT OF INTEREST**	None
**DATE REVIEW RETURNED**	14-Feb-2024

**GENERAL COMMENTS**	Thank you for inviting me to review this article.<br><br>I was interested to learn about the issues surrounding data quality pertaining to health inequalities in England given the scarcity of such data in Sri Lanka.<br><br>As an uninformed reader, I would have liked to have more background information in order to contextualize the analysis. For instance, I would have liked to know more about the health information systems (HIS) in place in England, which kinds of health inequalities are prioritized by them, who collects these data, what happens to them and, if known, the (potential) impact of them on health inequalities. Some discussion about the accessibility of these data and who can use them would also be helpful.<br><br>The analysis that appears under theme 1 looks like a list of data quality improving measures. It would be helpful to know how participants related them to improving data quality on health inequalities. Including a few quotes may be helpful in this section.

	Under theme 2, what kinds of data are referred to under issues that impact data collection? Would it be possible to provide specific examples or better contextualize the quotes? These issues would be very different based on the type of organization, health information system, etc. It would seem that some organisations collect certain kinds of data while others do not – perhaps these questions could be addressed by providing more background information in the introduction?<br><br>Lastly, what about the politics surrounding which kinds of inequalities are prioritized over others – do they influence efforts to improve data quality on health inequalities? The article seems to present the issue as a highly technical one devoid of politics. In other words, could health inequalities be addressed simply by improving data quality? Perhaps this could be discussed in the limitations section.<br><br>Other minor suggestions:<br>1) The title could be more interesting/punchy<br><br>2) The abstract refers to "creating a shared understanding around health inequalities" – the results stated before (in the abstract) need to convey the lack of such an understanding. Also, note the results appearing before that sentence make no reference to health inequalities. Indeed, the abstract says very little about health inequalities, including in the objective.<br><br>3) Methods<br>p. 5 line 47 – data quality "on health inequalities"?<br>P.7 lines 24 – What does "to recruit relevant and, where appropriate, representative samples" mean? How did you do that? Given the nature of the topic, specifying this would be important.<br>p.7, line 31 – What does "Participant eligibility was ascertained by personal communication prior to data collection" mean? Explain.<br>p. 7, line 55 – What does "knowledge of health inequalities" refer to? How was this measured?<br>In qualitative research, it is customary to reflect on researcher interests and motivations. The article states that the SRQR checklist was employed, but the researchers say nothing about their social locations or interest in the topic.<br><br>4) Analysis<br>p.9 lines 10-14 – Would it make sense to link the themes to health inequalities?<br>5) Strengths and limitations<br>Some of the strengths seem basic to qualitative research – do they merit mention? (e.g., open-ended interviewing, data coding etc.).<br>6) Conclusion - a new point about case studies appears here. This could be referenced in the discussion as well.<br>7) References – I am unable to assess whether the references are up to date and complete because I know very little about this literature as it pertains to the UK. I hope others with more expertise in the area will offer feedback on them.

VERSION 1 – AUTHOR RESPONSE

Reviewer: 1

Comments to the Author:
The paper provides some relevant analysis of some key issues that are relevant to data concerned with inequalities. The discussion highlights some important recommendations for policy and practice.

We thank the reviewer for their review and are pleased that they feel it highlights important recommendations.

1. The Title of the study focusses on resources, but some of the issues identified are not resources but processes i.e. communication. Therefore could the title be changed resource and processes.

We have implemented this change to the title.

2. On page 4, it states that 'In addition, data that are available are not always used to the best extent'. This needs to be referenced.

Thank you, we have added the following reference Ponce NA, Shimkhada R, Adkins-Jackson PB. Making Communities More Visible: Equity-Centered Data to Achieve Health Equity. Milbank Q 2023;101(S1):302-32. doi: 10.1111/1468-0009.12605

3. On page 7, you say that the tried to recruit a representative sample. However, there is no indication of what the sample is representative of. This needs to be made clearer.

We were aiming to recruit individuals working across the health and social care and who are involved in investigating health inequalities, and therefore representative of different roles. We have now added this to the methods.

4. On page 6, it states that the interviews were conducted via Zoom, but no rationale is provided for this.

Interviews were conducted online as it provided greater time and location flexibility for participants. In the participant information sheet, participants were offered in-person or on-line options. All participant opted for online. Zoom is a commonly used software for this purpose. We have indicted this in the methods.

5. On page 7, it says that the interviews were undertaken by 2 members of the research team. This needs to clearly state if by this is meant 2 members together, or 2 members individually, and if the former, the reason for this.

We have clarified that this was two members together. This was to reduce bias and ensure robustness in data collection.

6. On page 9, it states that an inductive approach to data analysis was used, however, closer analysis suggests that it was actually Inductive/Deductive Hybrid Thematic Analysis (as set out here: https://journals.sagepub.com/doi/10.1177/15586898221126816), which was undertaken. This needs to be clarified.

Thank you for highlighting this, we have now clarified that a deductive approach was used to code data and an inductive approach used to identify themes.

7. The Table of participants needs to be moved to where the sample frame is discussed as this is not results. Additionally, on page 8 where you say working across health and social care, this needs to be more specific if it refers to public sector health and social care organisations, or both.

Thank you for the suggestion regarding the Table of participants. We feel that it is more suited in the current section under results as it provides descriptive information about those who participated in the study. We have clarified the sentence on pg6 to “Participants included in the study were professionals that had a role in public sector health and social care, public health, or third sector organisations in England”.

8. There are variations of thematic analysis, and it would be good for the specific TA approach used to be specified and rationalised.

Please see response 6 which I think overlaps with this comment and has hopefully been addressed.

9. It would be good to know what the role of the participant was without a knowledge of health inequalities. For example, if this refers to the data analyst, then this could be significant in itself.

We do see the value in knowing the roles of those who did not have knowledge of health inequalities. But given the small number of study participants and this was one participant, we feel that providing this extra information may risk disclosure.

10. The tables presents for Analysis of Themes 1 and Themes are clear and detailed, but they do not particularly represent thematic analysis, they are more representative of quasi-statistical analysis, and this needs to be acknowledged and highlighted in the section on data analysis techniques.

Thank you for pointing this out. We have now acknowledged this in the data analysis section by stating that we have plotted the frequencies of theme elements.

11. Linked to this is the fact that these themes seem to indicate that either some form of quantitative data collection took place, or that the interviews had a highly structured element. This needs to be made clearer.

As noted above we did not carry out statistical analysis, but rather summarised key findings in tabular format to enable communications of key elements from the interviews, without overusing quotes. We have attempted to make this clearer with text changes to the analysis section and in the results.

12. Data usage barriers - should this be subdivided in the first part as Data Input issues?

Thank you for this suggestion, we have implemented this change and amended the plot.

13. In general, the participant's quotes are used very minimally, which is the essence of qualitative research. For example, Data usage barriers needs more validation of data through the use of participant's quotes. This needs to be reconsidered throughout the paper.

We thank the reviewer for this comment. We have reconsidered the use of quotes throughout. It was not always practically possible to use block quotes due to pauses and re-phrases from participants. Therefore, quotes are sometimes used in-text, this was done in order to create a free-flowing analysis of the results (like in this study).

14. Quote on page 19 is free hanging, needs some linkage.

Thank you for highlighting this, we have now hopefully linked it better to the text.

Reviewer: 2

I was interested to learn about the issues surrounding data quality pertaining to health inequalities in England given the scarcity of such data in Sri Lanka.

We are glad this manuscript was of interest.

As an uninformed reader, I would have liked to have more background information in order to contextualize the analysis. For instance, I would have liked to know more about the health information systems (HIS) in place in England, which kinds of health inequalities are prioritized by them, who collects these data, what happens to them and, if known, the (potential) impact of them on health inequalities. Some discussion about the accessibility of these data and who can use them would also be helpful.

We have tried to address this gap and added some clarifying sentences. As we note, the health data landscape in the UK is complex and data that can be used to monitor and address health inequalities can come from a variety of sources. Furthermore, priorities may vary from local, national and regional perspectives. Providing further details on the landscape of available data and access is outside the scope of this article and would considerably increase the word count. References 5, 6 and 10 provide an overview.

The analysis that appears under theme 1 looks like a list of data quality improving measures. It would be helpful to know how participants related them to improving data quality on health inequalities. Including a few quotes may be helpful in this section.

Thank you for the suggestion we included some explanatory sentences and have included a few quotes.

Under theme 2, what kinds of data are referred to under issues that impact data collection? Would it be possible to provide specific examples or better contextualize the quotes? These issues would be very different based on the type of organization, health information system, etc. It would seem that some organisations collect certain kinds of data while others do not – perhaps these questions could be addressed by providing more background information in the introduction?

As we discuss in the introduction the data that are collected that can be used to investigate health inequalities can be those relating to protected characteristics as well as socio-economic factors and geography. All of these data are impacted by the level of granularity with which they are collected across organisations involved in providing health and care, and the degree to which they can be shared. As also noted in the introduction, the health data landscape in the UK is complex. We were unable to within the scope of this document describe this landscape in detail but have provided a reference which does this.

The discussion under these quotes also provides description of the specific data they relate to.

Lastly, what about the politics surrounding which kinds of inequalities are prioritized over others – do they influence efforts to improve data quality on health inequalities? The article seems to present the issue as a highly technical one devoid of politics. In other words, could health inequalities be addressed simply by improving data quality? Perhaps this could be discussed in the limitations section.

Thank you for this discussion. We agree with the reviewer that politics does play a part in efforts to address health inequalities and improvements in data quality alone are unlikely to resolve differences in health outcomes. The aim of this study was not to investigate factors that impact on understanding and addressing health inequalities, but needs of health and care professionals in terms of resources to help support efforts to improve data quality. Nevertheless, we have added a line in the conclusion to acknowledge that efforts to improve data quality are in and of themselves unlike to address health inequalities.

Other minor suggestions:
1) The title could be more interesting/punchy

We have constructed the title needed to conform to the journal specifications which stipulate that the article title should include the study design and research question.

2) The abstract refers to “creating a shared understanding around health inequalities” – the results stated before (in the abstract) need to convey the lack of such an understanding. Also, note the results appearing before that sentence make no reference to health inequalities. Indeed, the abstract says very little about health inequalities, including in the objective.

Thank you for noting this omission which has now hopefully been resolved. We have indicated clearly that when we talk about data quality this is in relation to data used to investigate health inequalities.

3) Methods
p. 5 line 47 – data quality "on health inequalities”? – this has been clarified
P.7 lines 24 – What does “to recruit relevant and, where appropriate, representative samples” mean? How did you do that? Given the nature of the topic, specifying this would be important.

Thank for this, it has been bought up by reviewer 1 and has been addressed above.

p.7, line 31 – What does “Participant eligibility was ascertained by personal communication prior to data collection” mean? Explain.

As set-out in the methods our approach to recruitment was to compile a list of individuals who might be eligible in collaboration with our service partners. We followed this up with personal communication to better understand if individuals could and would be willing to contribute to the study. We have now clarified this with additional text in the manuscript.

p. 7, line 55 – What does “knowledge of health inequalities” refer to? How was this measured?
In qualitative research, it is customary to reflect on researcher interests and motivations. The article states that the SRQR checklist was employed, but the researchers say nothing about their social locations or interest in the topic.

Thank you for this note. Knowledge of health inequalities was not measured. However, as part of the interviews, participants were asked to talk about their awareness of inequalities in health and care. This was to enable a better understanding of participants' backgrounds. As stated in the results, all but one participant stated they had an understanding of the topic. However, all participants did acknowledge that given the breadth of field of health inequalities, perspective can vary.

The team involved in this work consisted of academic researchers (SM, LL and CB) as well those involved in the service sector in the East of England (SE, EO). This collaborative approach was to enable us to better understand differing perspectives. We have added this to the manuscript.

4) Analysis
p.9 lines 10-14 – Would it make sense to link the themes to health inequalities?

We have not added to avoid repetition and for the sake of brevity.

5) Strengths and limitations
Some of the strengths seem basic to qualitative research – do they merit mention? (e.g., open-ended interviewing, data coding etc.).

Thank you for making the above point. As this may be read by those not familiar to qualitative research, we feel while basic it is important to leave in the manuscript.

6) Conclusion - a new point about case studies appears here. This could be referenced in the discussion as well.

Thank you we have now referenced this in the discussion as well.

7) References – I am unable to assess whether the references are up to date and complete because I know very little about this literature as it pertains to the UK. I hope others with more expertise in the area will offer feedback on them.

VERSION 2 – REVIEW

**REVIEWER NAME**	Sealey, Clive
**REVIEWER AFFILIATION**	University of Worcester, Allied Health and Community
**REVIEWER CONFLICT OF INTEREST**	None
**DATE REVIEW RETURNED**	16-Apr-2024

**GENERAL COMMENTS**	I feel that the reveiosn made have imrpoved the paper. There is just oneminor amnedment required as below.<br><br>1. In relation to the Methods, you have written the following in the Response, but this may be more appropriate in the text:<br>Methods Interviews were conducted online as it provided greater time and location<br>flexibility for participants. In the participant information sheet, participants were<br>offered in-person or on-line options. All participant opted for online

**REVIEWER NAME**	Kumar, Ramya
**REVIEWER AFFILIATION**	University of Jaffna, Community and Family Medicine
**REVIEWER CONFLICT OF INTEREST**	I have no competing interests to declare.
**DATE REVIEW RETURNED**	21-Apr-2024

**GENERAL COMMENTS**	The paper reads very well now.<br>Three minor comments:<br>p. 13 – an example of over aggregation would be helpful to better understand the consequences.<br>p. 14 – there appears to be a typo in the theme 3 subtitle.<br>'Data' is used in singular form in most places. I'm not sure whether the plural form was used intentionally in others ('data were').

VERSION 2 – AUTHOR RESPONSE

Reviewer: 1
Dr. Clive Sealey, University of Worcester
Comments to the Author:
I feel that the reveiosn made have imrpoved the paper. There is just oneminor amnedment required as below.
We are pleased that the paper has improved and have made the suggested amendment below.

1. In relation to the Methods, you have written the following in the Response, but this may be more appropriate in the text:
Methods Interviews were conducted online as it provided greater time and location flexibility for participants. In the participant information sheet, participants were offered in-person or on-line options. All participant opted for online.
This is now included this in the methods.

Reviewer: 2
Dr. Ramya Kumar, University of Jaffna
Comments to the Author:
The paper reads very well now.
Three minor comments:
p. 13 – an example of over aggregation would be helpful to better understand the consequences.
We have included an example of ethnicity data and geography data as examples.

p. 14 – there appears to be a typo in the theme 3 subtitle.
'Data' is used in singular form in most places. I'm not sure whether the plural form was used intentionally in others ('data were').

As requested by the editor we have now competed a proof-read and made sure to check on the above point.
